# Giant Fibroelastoma of the Aortic Valve

**DOI:** 10.1155/2013/754235

**Published:** 2013-09-04

**Authors:** Michele di Summa, Federica Iezzi

**Affiliations:** Division of Cardiac Surgery, Spitali Europian-GVM Care & Research, P.O. Box 1051, Autostrada Tiranë-Durrës, Qafa e Kasharit, tek Mbikalimi për Rinas, 1051 Tiranë, Albania

## Abstract

Fibroelastomas account for less than 10% of all cardiac tumours, representing the most common valvular and the second most common cardiac benign tumour, following myxomas. Fibroelastomas are histologically benign; they can result in life-threatening complications such as stroke, acute valvular dysfunction, embolism, ventricular fibrillation, and sudden death. Surgical resection should be offered to all patients who have symptoms and to asymptomatic patients who have pedunculated lesions or tumors larger than 1 cm in diameter. Valve-sparing excision produces good long-term results in most instances. We report our surgical experience of a giant fibroelastoma in the aortic valve.

## 1. Introduction

Cardiac fibroelastomas are the most common benign neoplasms of the cardiac valvular structures.

Fibroelastoma is often attached to valve leaflets, most often to the aortic valve, and less frequently to the tricuspid, mitral, and pulmonary valves. Although most cases of fibroelastoma are incidental findings because they are asymptomatic, some show a strong propensity toward embolization, causing angina, myocardial infarction, transient ischemic attack, stroke, or sudden death when the tumor is in the left side of the heart.

Here, we report our treatment of a giant aortic valve fibroelastoma.

## 2. Case Report

An 18-year-old man was referred for the evaluation of fatigue, chest pain, and syncope.

His fatigue initially consisted of effort intolerance. He did not initially seek medical attention for his fatigue and syncope. His chest discomfort was described as a squeezing sensation that was substernal in location without radiation to any other position. His syncope was always preceded by substernal chest pressure, dizziness, and breathlessness.

A transesophageal echocardiogram demonstrated a 70 mm mobile mass adherent to the left coronary cusp of the aortic valve, sign of left ventricular outflow tract obstruction.

The mass was a pedunculated, echo dense, stipple in texture structure, with well-demarcated borders, features typical of a fibroelastoma.

The cardiac fibroelastoma was responsible for left ventricular outflow tract obstruction and valvular dysfunction.

The patient underwent surgical excision of the mass.

Preparation for operation, median sternotomy, cardiopulmonary bypass, and myocardial management were performed. The tumor was found to have a wide attachment to the surface of the ventricular aspect of the left coronary cusp extending from the aortic annulus to the edge of the cusp.

**Figure 1:**
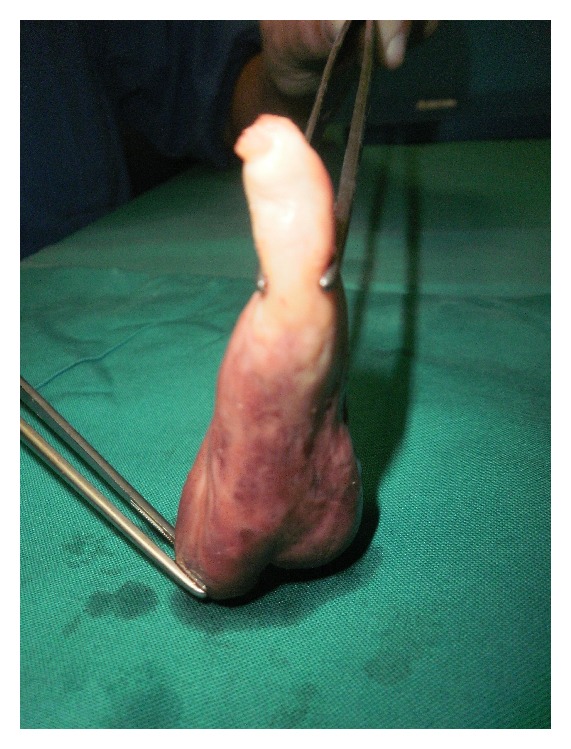
Giant aortic fibroelastoma.

Tumor had well-demarcated borders and homogenous texture in appearance (Figure 1).

**Figure 2:**
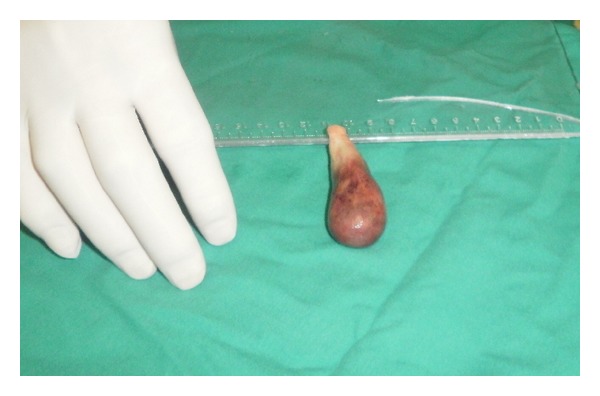
Fibroelastoma after surgical excision.

The size of the tumor was about 70 mm × 20 mm (Figure 2).

The tumor in the chamber had independent mobility on valvular endocardium surface and has an identifiable stalk.

Aortic valve insufficiency was due to the delay of diagnosis and following the mechanical trauma of the valve.

A valve sparing technique with simple shave excision of the tumour was undertaken with particular care in avoiding embolization and ensuring that no remnants from fragmentation of the tumour were left behind both locally on the cusp and in the vicinity of ascending aorta and left ventricle.

Aortic valve competence was confirmed intraoperatively with transesophageal echocardiographic approach before the aortotomy was closed.

Pathological examination of the excised mass showed a frond-like villous surface with a hyalinized myxoid core, delineated by a layer of endothelial cells—findings consistent with a classic fibroelastoma.

The patient recovered without any complications and remained free of symptoms.

No mass was detected by echocardiography in the patient during the 6-month follow-up period.

## 3. Discussion

Cardiac fibroelastomas have a high propensity to affect the aortic valve, the left ventricular outflow tract, and the anterior mitral leaflet.

Fibroelastomas arising from semilunar valves are located with equal frequencies on the ventricular and arterial sides of the valves. In addition, nonvalvular origin was observed in approximately 16% cases that included left and right ventricular septal and mural endocardial surfaces, atrial endocardium, papillary muscles, chordae tendineae, or intima of the right coronary ostium [1].

The majority of patients are asymptomatic and the tumours are most often incidentally diagnosed. Fibroelastomas have the potential to lead to life-threatening complications from embolization of fragments into the coronary arteries, systemic circulation and pulmonary circulation, and left ventricle outflow tract obstruction [2].

Symptomatic patients should be referred for curative surgical excision of the tumour. Asymptomatic patients with large (>1 cm) mobile masses, especially left sided, should also be considered candidates for curative surgical excision due to the increased risk of cardiovascular complications from embolization and sudden cardiac death. Asymptomatic patients with small left-sided nonmobile lesions are being closely followed up with echocardiography until symptoms develop or tumours enlarge and become mobile [3].

Aortic valve should be preserved preferably. In case there is resultant valve defect, it should be repaired; otherwise a valve replacement is warranted. The surgical resection is curative, safe, and well tolerated [4].
